# Rapid Assessment of Environmental Health Impacts for Policy Support: The Example of Road Transport in New Zealand

**DOI:** 10.3390/ijerph13010061

**Published:** 2015-12-22

**Authors:** David Briggs, Kylie Mason, Barry Borman

**Affiliations:** 1 Emeritus Professor, Department of Epidemiology and Biostatistics, Imperial College London, London W2 1PG, UK; 2 Centre for Public Health Research, Massey University, Wellington 6140, New Zealand; k.mason@massey.ac.nz (K.M.); b.borman@massey.ac.nz (B.B.)

**Keywords:** health impact assessment, road transport, New Zealand, environmental burden of disease, road accidents, air pollution, traffic noise, physical activity

## Abstract

An integrated environmental health impact assessment of road transport in New Zealand was carried out, using a rapid assessment. The disease and injury burden was assessed from traffic-related accidents, air pollution, noise and physical (in)activity, and impacts attributed back to modal source. In total, road transport was found to be responsible for 650 deaths in 2012 (2.1% of annual mortality): 308 from traffic accidents, 283 as a result of air pollution, and 59 from noise. Together with morbidity, these represent a total burden of disease of 26,610 disability-adjusted life years (DALYs). An estimated 40 deaths and 1874 DALYs were avoided through active transport. Cars are responsible for about 52% of attributable deaths, but heavy goods vehicles (6% of vehicle kilometres travelled, vkt) accounted for 21% of deaths. Motorcycles (1 per cent of vkt) are implicated in nearly 8% of deaths. Overall, impacts of traffic-related air pollution and noise are low compared to other developed countries, but road accident rates are high. Results highlight the need for policies targeted at road accidents, and especially at heavy goods vehicles and motorcycles, along with more general action to reduce the reliance on private road transport. The study also provides a framework for national indicator development.

## 1. Introduction

In the modern, highly interconnected and technologically complex world, many of the issues faced by policy-makers are themselves interconnected and complex. Assessing these problems, and devising suitable policy responses, thus faces many challenges and requires suitably expansive methods of analysis. One way of conducting these analyses is through the use of integrated assessments. In recent years, a number of such assessments have been carried out, mainly focusing on systemic environmental issues such as climate change, energy technologies or regional land use change [1,2,3]. Few of these have been designed to assess impacts on human health. In order to redress this deficiency, therefore, the EU-funded INTARESE and HEIMTSA projects developed the concept of integrated environmental health impact assessment (IEHIA) [4]. Building on the environmental burden of disease [5] approach, this aimed to assess policy issues by tracking all the relevant agents of impact, along all their main pathways from source to exposure, and thereby deriving estimates of the cumulative health impact, together with the proportion of that impact attributable to each specific source.

Conducting full, integrated assessments of this sort is far from easy. The processes and impacts of concern are often only partially understood, relevant data are often incomplete, and knowledge and expertise in the various discipline areas may be limited or difficult to bring together for the purpose of an assessment. Unsurprisingly, therefore, the majority of integrated assessments carried out to date have been done in richer, developed countries, especially in North America and Europe. Elsewhere, policy-makers are understandably cautious about their capacity to carry out assessments successfully, and the costs of doing so. Equally, for many strategic applications—such as developing national environmental health action plans or environmental health indicators—it may be difficult to justify such assessments, or to incorporate them into the often short timescale for policy formulation.

In many contexts, therefore, there is a need for simpler, low-cost and/or more rapid assessments, that can used within the constraints of available data, expertise and time. Allowance for such assessments is, in fact, recognised in the IEHIA framework [4], where they are used as a way of scoping and screening, to determine whether more complete assessments are worthwhile and, if so, to define their scope and content. Precedents for rapid assessments also exist in the fields of both environmental impact assessment (EIA) and health impact assessment (HIA). In the context of EIA, various guidelines and toolkits for rapid assessment have been developed (e.g., [6]), while in a survey of HIA, Davenport *et al*. [7] reported that over half the studies they considered comprised what the authors described as rapid assessments. Many of these, however, are essentially qualitative in approach, and in the field of HIA the term rapid assessment (or appraisal) is sometimes restricted to studies comprising a short workshop with stakeholders or experts [8]. Many are also local in scope [7,9]. More wide-ranging, quantitative studies seem to have been rarely attempted.

This paper develops and applies a rapid assessment methodology to quantify health impacts of road transport in New Zealand. Its aims are:

1. To demonstrate the feasibility of undertaking simple, speedy assessments of complex policy issues within the context of limited data and prior knowledge;
2. To outline some of the methods and approaches that can be applied in this context, including simple approximation and modelling techniques;
3. To identify and estimate the scale of uncertainties that arise in the assessment, and compare the results with those from other studies both within New Zealand and overseas;
4. To discuss the implications of the results of the assessment for national policy on road transport and associated indicator development in New Zealand.

The work presented here has been undertaken in conjunction with the national Environmental Health Indicators programme, funded by the New Zealand Ministry of Health.

## 2. The Context

New Zealand is a relatively small and sparsely populated country, with a land area of 271,000 km^2^ and a population of 4.47 million people (2013 Census). As in other developed countries, road transport plays a major role both economically and socially, and transport policies account for a significant proportion (*ca.* 10 per cent) of total government spending [10]. The road network is about 94,000 km in length, of which about 11,000 km are classified as highways, and about 32,000 km are unsealed [11]. Private cars account for the large majority of passenger transport: between 2010 and 2014, 79 per cent of travel time was undertaken as a car driver or passenger, with about 0.4 per cent by motorcycle, and 4.1 per cent by public transport (bus, rail, ferry), the majority by bus [12]. The remainder is by foot and bicycle. Reflecting these statistics, vehicle registrations per head of population are among the highest in developed countries with about 600 private cars per 1000 people in 2011 [13]. Roads also accounted for about 82 per cent of total land freight (in tonnes-km) in 2012 [14].

Given the spread of the road network, and the traffic volumes it carries, it may be expected that road transport in New Zealand has significant effects on human health, though no systematic and comprehensive assessment of these impacts have yet been made. Police data on road accidents are, however, routinely collated by the relevant agencies (Ministry of Transport and New Zealand Transport Agency) and reported both as part of national and international indicator series [15,16]. Three assessments have also been made (in 2001, 2006 and 2012) of the health effects of air pollution exposures from different source activities, and these have suggested a major contribution from the road transport sector [17,18,19]. Additionally, Lindsay *et al*. [20] estimated the effects of moving short urban car trips (≤7 km) to cycling in New Zealand, and examined impacts via air pollution, accidents and physical activity. Their results indicated substantial savings in the numbers of deaths annually, depending on the degree of reduction in vehicle kilometres travelled. At the same time, initiatives are being pursued by the ministries of Health, Environment and Transport to develop new indicator sets for policy support, all of which include transport-related impacts. Together, these studies highlight the need for a more comprehensive assessment and monitoring of transport-related health impacts, and motivated the study carried out here.

## 3. Methods

### 3.1. Scoping and Framework

#### 3.1.1. Conceptual Model

The first stage in conducting an IEHIA is to scope the issue of concern and develop a conceptual model that spells out all the sources, agents, pathways and impacts that might need to be considered, and the links between them [4]. In a full assessment, this should ideally be done in association with all the major stakeholders; for the purpose of the rapid assessment conducted here, it was carried out by the study team.

**Figure 1:**
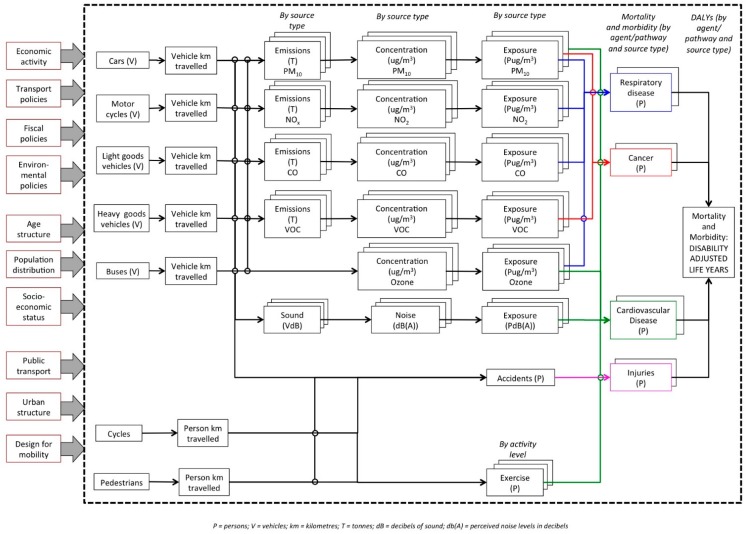
Scoping of the road transport-health system.

As a basis for conceptualisation, an initial review of statistics and indicators on road transport in the country was undertaken, aimed at establishing the key characteristics of the system under study. This drew especially on data reported by the New Zealand Transport Agency (which is responsible for road planning, management and maintenance) and the Ministry of Transport (which is responsible for transport policy and regulation). A brain-storming and mind-mapping session was then undertaken by the authors, and a draft conceptual model devised, summarising the main transport sectors, impact pathways and health outcomes of interest. Subsequently, this was refined by reference to past studies and assessments of transport-related health impacts [9,21,22,23,24,25], and the adjusted model presented in the form of a system diagram. This was then used as the framework for the assessment. Further, minor adjustments were made as the assessment progressed to reflect new insights gained from the analysis (e.g., definition of buses as a separate transport mode). Figure 1 shows the final, amended version of the model.

#### 3.1.2. Identifying Relevant Exposures and Health Impacts

The model identified four main pathways for health impacts: traffic accidents, air pollution, road traffic noise and physical activity. For each pathway, the main exposures and health effects were defined and evaluated in terms of their evidence base, through a survey of the relevant literature.

The role of road traffic in causing physical injuries is evident and well-documented and is already a major focus of policy concern. Data are also readily available through routine monitoring and reporting.

Health effects associated with air pollution are likewise relatively clearly defined, and effects have been measured for a range of outcomes and pollutants. Most attention has focused on the risks associated with particulate matter measured in various ways (e.g., as PM_10_, PM_2.5_, ultrafines, elemental carbon, black smoke), and nitrogen dioxide. In the case of particulate matter, well-established effects have been reported for chronic respiratory and cardiovascular diseases and some cancers, as well as low birth weight, mainly in relation to fine particulate matter (PM_2.5_) [26,27,28,29]. Evidence is less robust for PM_10_, but several cohort studies have shown impacts on all-cause mortality [30]. Short-term effects have also been widely reported for particulates, measured in various ways, and both mortality and hospitalisation for a range of outcomes [31]. Nitrogen dioxide has mainly been implicated in acute respiratory effects (especially asthma in children). For long-term exposures, evidence is more uncertain, but independent effects have been reported for all-cause mortality and, in a large recent study, with new onset of asthma in adults [27,32].

The health effects of exposure to excessive road traffic noise are more contentious and have received less attention than those associated with traffic-related air pollution or air traffic noise. It has also been suggested that the associations between adverse health outcomes and traffic noise might be partially confounded by effects of air pollution [33,34]. Nevertheless, several studies have shown seemingly independent effects of road traffic noise on raised blood pressure and chronic heart disease [34,35,36,37], and on this basis road traffic noise was included in this assessment. Night-time noise is considered to be the most influential in the case of these effects, and reflecting this L_den_ (weighted noise levels during the day, evening and night-time periods) is often used as the main exposure indicator in health studies.

The role of road transport in influencing levels of physical activity has received increasing attention in recent years, especially as the problem of the so-called obesity epidemic has been recognised [21,25]. Urban design, and road transport more specifically, play a powerful role in determining people’s level of physical activity in two main ways: by influencing the choice of transport mode for routine journeys such as travelling to work or school, and more indirectly by affecting the walkability and cycleability of the residential environment, and thus recreational choices. Attributing lack of physical activity entirely to road transport is, of course, inappropriate: transport is only one of a nexus of factors that affect activity choices and patterns. However, short journeys can be done by active means, and in environments where the appropriate infrastructure is in place and a culture of walking and cycling has been fostered, this accounts for a large proportion of intra-urban trips, with consequent health benefits [38,39]. Lack of physical activity, on the other hand, is an important risk factor for human health, and sedentary lifestyles and lack of regular exercise have been shown to increase the risk of raised blood pressure, cardiovascular and respiratory diseases, some cancers and all-cause mortality [40,41,42].

Based on this review of the evidence, the following pathways and outcomes were included in this assessment: traffic deaths and injuries due to road accidents; all-cause mortality from particulate matter (PM_10_) and nitrogen dioxide; effects of road traffic noise on cardiovascular disease; and effects of physical inactivity on cardiovascular disease, diabetes, colon cancer and breast cancer. Several other possible pathways and outcomes were rejected because of lack of firm evidence of the relative risks, and/or detailed exposure or health data. These included air pollutants such as fine particulate matter (PM_2.5_), ozone, carbon monoxide, black smoke, sulphur dioxide and elemental carbon, short-term effects of particulates and nitrogen dioxide, and effects of road traffic noise on quality of life, sleep loss, cognition and subclinical symptoms of stress. Health impacts due to climate change induced by emissions from road traffic were also excluded, even though these may be substantial [23,43], because of the difficulties involved in estimating New Zealand’s share of these global impacts.

### 3.2. Overview of Approach to Estimating Health Burden

The assessment carried out here is diagnostic [4] in that it attempts to quantify, and attribute to source, health impacts arising from road transport in New Zealand. The assessment thus uses an implied counterfactual scenario of no road transport. It should be noted that for road accidents and noise, this implies a zero exposure; for the other risk factors, the exposures are non-zero.

For this analysis, an environmental burden of disease approach was employed, using comparative risk assessment methods. These methods are based on the concept of the population attributable fraction (PAF), or potential impact factor (PIF) for cases with non-zero counterfactual exposures [44]. PAF represents the proportion of health events attributable to a specific risk factor. It is calculated by: (i) estimating the level of exposure (and numbers exposed) to that risk factor; (ii) selecting appropriate exposure-response functions for the risk factor from the literature [45]; (iii) applying the relative risks to the estimated exposures to derive the population attributable fraction. These attributable fractions are then applied to data on deaths and disability-adjusted life years (DALYs), to estimate the health burden attributable to each risk factor.

We estimated the attributable deaths, years of life lost (YLLs) and healthy years of life lost (DALYs) due to road transport for each pathway. Data were sourced for the year 2012 or, where these were lacking, the nearest available year.

### 3.3. Exposure-Response Functions

For the selected exposures and health outcomes, we used meta-analyses and burden of disease methodology reports to identify the exposure-response functions and relative risks (Table 1).

**Table 1:** Exposure-response functions for selected health effects from road transport.

Exposure	Health Outcome	Age Group	Exposure—Response Function/Relative Risk (RR)	Health Data	Source
Particulate matter (PM_10_)	All-cause mortality	30+ years	1.07 (1.03–1.10) per 10 µg/m^3^ annual average	All-cause mortality excluding external causes (V00–Y98)	Hales *et al*. [46]
		Infants (1 month to 1 year)	1.05 (1.02–1.08) per 10 µg/m^3^ annual average	All-cause mortality excluding external causes (V00–Y98)	Lacasana *et al*. [47]
Nitrogen dioxide	All-cause mortality	30+ years	1.055 (1.031–1.08) per 10 µg/m^3^ annual average, for levels above 20 µg/m^3^	All-cause mortality excluding external causes (V00–Y98)	Atkinson *et al*. [48]
Road traffic noise	Ischaemic heart disease	30+ years	1.046 (1.015–1.079) per 10 dBA increase in L_den_ above 48 dBA	ICD10: I20–I25	Vienneau *et al*. [49]
	Stroke	30+ years	1.014 (0.964–1.066) per 10 dBA increase in L_den_ above 48 dBA	ICD10: I60–I64 (excluding I63.6)	Vienneau *et al*. [49]
	Hypertensive diseases	30+ years	1.076 (1.032–1.121) per 10 dBA increase in L_den_ above 48 dBA	ICD10: I10–I15	Vienneau *et al*. [49]
Physical activity ^1^	Ischaemic heart disease	30–64 years	High: RR = 1.00	ICD10: I20–I25	Danaei *et al*. [41]
			Moderate: RR = 1.15		
			Low: RR = 1.66		
			Inactive: RR = 1.97		
	Ischaemic stroke	30–64 years	High: RR = 1.00	Stroke: ICD10: I60–I69 (then applied 36%)	
			Moderate: RR = 1.12		
			Low: RR = 1.23		
			Inactive: RR = 1.72		
	Breast cancer	30–64 years	High: RR = 1.00	ICD10: C50	
			Moderate: RR = 1.25		
			Low: RR = 1.41		
			Inactive: RR = 1.56 (for 30–44 years); 1.67 (for 45–64 years)		
	Colon cancer	30–64 years	High: RR = 1.00	ICD10: C18	
			Moderate: RR = 1.07		
			Low: RR = 1.27		
			Inactive: RR = 1.80		
	Diabetes mellitus (type 2)	30–64 years	High: RR = 1.00	ICD10: E11	
			Moderate: RR = 1.21		
			Low: RR = 1.50		
			Inactive: RR =1.76		
Traffic injuries	Deaths and injuries from road transport	All ages	100% attributable	Road transport injuries from ICD10: V00–V89	

Note: ^1^. Physical activity levels are defined as follows: high = 1+ h per week of vigorous activity and 1600+ MET-minute per week (MET = metabolic equivalent of task, *i.e.*, equivalised energy output); moderate = either 2.5+ h per week of moderate activity, or 1+ h per week of vigorous activity and 600+ MET-mins per week; low = less than 2.5 h per week of moderate activity or less than 600 MET-min per week; inactive = no moderate or vigorous physical activity per week.

### 3.4. Data and Analysis

Where possible we used existing data on the population exposed and health outcomes to assess health impacts. Where data were unavailable (specifically for nitrogen dioxide and noise), approximation methods were employed to estimate exposures, as outlined below.

#### 3.4.1. Traffic Injuries

Data on road accidents in 2012 were obtained from the annual report of the national Ministry of Transport [50]. This report gives numbers of fatal and non-fatal injuries, by transport mode and age group for each calendar year. We included deaths due to road transport by transport mode.

#### 3.4.2. Particulate Matter

In New Zealand, data on exposures to particulate matter are limited, with regulation and air pollution monitoring focussing on PM_10_. Assessing health impacts from particulate matter thus requires either using the less reliable exposure-response functions for PM_10_, or converting PM_10_ data to estimates of PM_2.5_. A further difficulty in New Zealand is that domestic wood-burning fires are still common throughout the country and substantially contribute to local atmospheric particulate concentrations. Measured PM_10_ concentrations, therefore, do not provide a direct indication of exposures from traffic sources.

For this assessment, we used the published estimates of attributable premature deaths due to long-term PM_10_ exposure from the HAPINZ (Health and Air Pollution in New Zealand) study, which was recently updated for 2012 data [19]. The HAPINZ study used purpose-designed regression models to derive estimates of average annual concentrations of PM_10_ for small areas across the country, and estimated the attributable mortality based on New Zealand exposure-response functions [46]. On the basis of source apportionment using regression methods, the HAPINZ study suggested that motor vehicles accounted for 22 per cent of anthropogenic health impacts from PM_10_ in 2006 [18]; this proportion was applied to 2012 data to estimate attributable deaths due to transport-related air pollution.

#### 3.4.3. Nitrogen Dioxide

Although nitrogen dioxide is extensively monitored by local authorities in New Zealand, using passive samplers, concentration data are insufficient to provide reliable estimates of exposures across the population. Data on road traffic and emissions are also inadequate as a basis for country-wide modelling using atmospheric dispersion models. For this assessment, therefore, the passive sampling data (*N* = 150 sites) were first subdivided into five concentration categories. Discriminant analysis techniques were then used to develop a model that reliably distinguished between these categories on the basis of the road density, population density and mean altitude in the small area (meshblock) in which the sites lay. This model (74 per cent classification accuracy) was used to assign all other meshblocks in the country to exposure categories, and the resulting meshblock populations summed for each category. Traffic emissions are the main source of nitrogen dioxide in New Zealand [17], and based on analysis of the data in the HAPINZ study [17] and from the 150 monitoring stations used here, a background concentration of *ca.* 10–20 ug/m^3^ (mean 14 ug/m^3^) can be assumed for non-trafficked areas. This was therefore adopted as the exposure level under the counterfactual scenario. The exposure-response function recommended by the HRAPIE project [48] was then applied to both the modelled current and counterfactual scenarios to give the population attributable fraction in each exposure category; from these, the overall population attributable fraction and attributable burdens were calculated. The overall attributable burden was reduced by 33% as recommended by Atkinson *et al*. [48] to account for the potential overlap with PM_10_ effects.

#### 3.4.4. Road Traffic Noise

Monitoring of ambient noise is not undertaken in any systematic way in New Zealand, and road noise is exempted from the noise regulations in the Resource Management Act 1991 [51], which provides local authorities with their environmental powers. There is no obligation on local authorities or the New Zealand Transport Agency to model noise levels associated with roads. Purpose-designed noise modelling was also not feasible for this assessment, as data on road traffic flows and characteristics at street or road-segment level are not readily available in a coherent, georeferenced form.

Approximation methods were therefore used to estimate exposure to road traffic noise in New Zealand. Assessment was confined to the five main cities—Auckland (population 1.5 million), Wellington (394,000), Christchurch (357,000), Hamilton (150,000) and Dunedin (124,000)—which together make up about 57% of the national population. These are considered to be the main areas potentially subject to significant traffic noise; other hotspots may occur in smaller towns and on some major highways (e.g., State Highway 1), but in general the affected population is likely to be small. For this reason no attempt was made to extrapolate the estimates to the rest of New Zealand.

Potential exposure of the resident population to road traffic noise in these areas was categorised on the basis of road class and distance from road. Three noise exposure categories were defined, a priori: high (living within 50 metres of a major highway), moderate (living within 50–200 metres of a major highway, 100 metres of a main road, or 50 metres of a secondary road), and low (other). Using GIS techniques, roads in the Land Information New Zealand (LINZ) 1:50,000 road centreline data were buffered at the required distances and overlaid onto address point data (spatial resolution *ca*. 1 metre). Census population counts for 2013 in each territorial authority (67 nationwide) were then apportioned according to the proportion of the address points within each buffer zone.

To estimate exposures in these zones, traffic count data were collated for the entire state highway network (*n* = 1920 sites), and for urban roads in Auckland (*n* = 3694), Wellington (*n* = 502) and Hamilton (*n* = 212). On the basis of these data, noise levels at the inner and outer limits for each zone were modelled using the TRANEX model [52], and the average taken. To represent low exposure zones, modelling was done for points 500 metres from the nearest highway and 400 metres from the nearest main or secondary road. For all analyses models were parameterised to represent indicative conditions: building frontages at 180 degrees to the road, no gradient, no barriers or vegetation cover, impervious road surfaces, and speeds defined by the regulatory speed limits. The resulting average (and standard deviation) noise levels were as follows: high 67.8 (4.9) dBA, moderate 60.6 (7.7) dBA, low 49.7 (6.5) dBA. These values were assigned to everyone resident within the associated exposure zone.

Exposure-response functions for ischaemic heart disease, stroke and hypertensive diseases due to road traffic noise [49,53] were used to calculate the population attributable fraction for each condition, for each exposure group and city. Results were then summed across cities to give the estimate of the overall health burden attributable to road traffic noise.

#### 3.4.5. Physical Activity

Levels of physical activity were derived from the 2006/07 New Zealand Health Survey [54]. This included the International Physical Activity Questionnaire (IPAQ) short form on self-reported physical activity (including both for leisure and work); data from the questionnaire had been categorised by the Ministry of Health as per the 2010 Global Burden of Disease Study methods [41] into inactive, low, moderate and high physical activity levels.

Assessments of health impacts of physical activity and inactivity associated with road transport were estimated for adults aged 30–64 years only. This reflects the circumstance that few commuters are over the age of 64, while the large majority of relevant health effects from physical activity occur in the 30+ age group—and it is for these that the most reliable exposure-response functions have been developed. The assessment also considered only commuting for work, since this represents a regular (daily) activity which thus implies long-term health effects. Work travel is also more closely associated with the road system than some other forms of travel (e.g., for recreation) which often take place off-road. Based on the counterfactual of no road transport, the assessment assumed that all adults who currently go to work mainly (*i.e.*, in terms of distance) on foot or by cycle had been raised from the low physical activity level to the moderate level as a result of their commute. Estimates of the numbers involved were made on the basis of trip data by mode and destination, derived from the New Zealand Household Travel Survey [12]. Annual numbers of trips to work were divided by 250 (to represent the approximate number of work days), in order to indicate the number of individuals travelling routinely by each mode.

Data on physical activity levels from 2006/07 were used as they were readily available, and overall levels of physical activity did not change substantially from 2006/07 to 2011/12 [55]. Relative risks for adults aged 30+ years were obtained from the Global Burden of Disease Study 2010 [41] for a range of health outcomes (ischaemic heart disease, ischaemic stroke, breast cancer, colon cancer and diabetes) (Table 1). For ischaemic stroke, data for all stroke (ischaemic and haemorrhagic) were used, with 36% of the total burden assumed to be due to ischaemic stroke, based on an analysis of stroke deaths for 20–64 years in high-income countries in 2010 [56].

#### 3.4.6. Health Data

Mortality data were sourced from published tables for 2012 [57], by cause and age group. For health effects from road traffic noise, the New Zealand Mortality Collection dataset for 2011 was analysed to estimate deaths for the five cities of interest. Years of life lost (YLLs) were calculated from the death data, using life expectancy weights by 5-year age group [54].

Because detailed data were not available on the severity or age of onset/occurrence of disease and non-fatal injuries, years lived with disability (YLD) could not be calculated directly. They were therefore imputed from the YLLs, by applying the YLD:YLL ratios from the Global Burden of Disease Study 2010 database for New Zealand [58], by specific health condition. The resulting estimates of YLD were added to the YLD to give disability adjusted life years DALYs. This method assumed that the ratio of fatal to non-fatal health loss had not substantially changed since 2010. The YLDs took into account disease severity (as they had been calculated using disability weights); following the approach used in the Global Burden of Disease Study 2010 [59], neither the YLD or YLL data were age-weighted or -discounted.

#### 3.4.7. Estimating Health Impacts by Transport Mode

Where possible, we estimated the health burden by transport mode: cars, light goods vehicles (LGV = light trucks, vans), heavy goods vehicles (HGV = heavy trucks), buses, motorcycles, cycling and walking. This involved the approximate allocation of deaths and YLLs by transport mode, according to the underlying cause (or modal source) of exposure.

For road transport accidents, separate listings are available for HGVs and motorcycles, and responsibility for the associated casualties were assigned accordingly. All other casualties (*i.e.*, those not involved in HGV or motorcycle accidents) were apportioned to cars, LGVs and buses according to their relative travel distance. Pedestrians and cyclists were thus treated as “innocent” victims in any traffic accidents in which they were involved.

For air pollution, allocation was approximate, as emissions estimates by mode are not available. Using emission factors derived from modelling in Auckland [60], and taking account of the national fleet composition (diesel and gasoline) and vehicle kilometres travelled, total PM_10_ emissions were roughly allocated as follows: 43% cars, 28% LGV, 28% HGV, 1% buses and 0.2% motorcycles. For nitrogen dioxide, the percentages were 45% cars, 15% LGV, 38% HGV, 1% buses and 0.4% motorcycles.

Lacking other means of allocating noise to source vehicle type, the attributable health burden from road noise was apportioned according to the total vehicle kilometres travelled by mode.

#### 3.4.8. Estimating Uncertainties

By their nature, health impact assessments are approximate and involve a range of judgements. Uncertainties arise at every stage in the analysis from the initial issue framing, through data collection and modelling, to the interpretation and reporting of the results [61,62]. Estimation of these uncertainties is thus an integral part of the assessment. In a rapid assessment such as this, detailed quantification of uncertainties is not possible, but *post hoc* sensitivity analyses were conducted to estimate the possible effects of uncertainties in the estimated impacts for particulates, nitrogen dioxide, noise and physical activity (see Appendix Table A2). Following the example of Forastiere *et al*. [63] and Knol *et al*. [64], a qualitative assessment of the uncertainties was also made for each of the main pathways, loosely based on the guidelines developed by Intergovernmental Panel on Climate Change [65]. Here, five levels of confidence in the estimates were applied: very high (implying that the estimates were considered to be within 10 per cent of the true value); high (10–20 per cent), moderate (20–50 per cent), low (50–100 per cent) and very low (>100 per cent).

## 4. Results

### 4.1. Attributable Health Burden from Road Transport

Table 2 summarises the results. Summing over all sources and pathways, the assessment suggests that road transport was responsible for a net burden of 610 premature deaths and 17,815 years of life lost (YLLs) in New Zealand in 2012. These deaths represented about 2.1 per cent of total annual mortality, and 3.3 per cent of total years of life lost in 2012. Overall, road transport led to an estimated 24,736 healthy years of life lost (DALYs) in 2012, which includes both the fatal and non-fatal health burden due to road transport.

Injuries caused by traffic accidents accounted for 308 deaths in 2012 (47 per cent of total deaths attributable to road transport). The relatively young age of victims in road traffic accidents (median *ca.* 40 years) means that the contribution to years of lost life is high (13,974 YLLs, 73 per cent of the attributable total). About 70 per cent of traffic accident deaths comprise drivers and passengers of cars, LGVs and HGVs, which dominate traffic volumes in New Zealand. Motorcycles make up 16 per cent of deaths, pedestrians 11 per cent and cyclists 3 per cent.

**Table 2:** Summary of estimated health impacts, 2012 (or latest year).

Agent/Pathway	Outcomes	Mode/Agent	Estimated Attributable Burden (2012)						
			Deaths (% of Total)		Years of Life Lost (YLLs) (% of Total)		YLD/YLL Ratio	DALYs (% of Total)	
Traffic injuries	Road transport injuries	Total road transport deaths	308	(47%)	13,974	(73%)		21,244	(80%)
		*Cars/HGVs*	217	(33%)	9990	(53%)	0.53	15,264	(57%)
		*Motorcycles*	50	(8%)	2202	(11%)	0.47	3231	(12%)
		*Pedestrians*	33	(5%)	1487	(8%)	0.53	2279	(9%)
		*Cyclists*	8	(1%)	296	(2%)	0.59	469	(2%)
Air pollution	All-cause mortality (30+ years)	Air pollution	283	(44%)	4449	(23%)	0	4449	(17%)
		*PM_10_*	218	(34%)	3426	(18%)		3426	(13%)
		*Nitrogen dioxide*	65	(10%)	1023	(5%)		1023	(4%)
Road traffic noise	IHD, stroke, hypertensive diseases (30+ years)	Noise from road vehicles	59	(9%)	821	(4%)		917	(3%)
		*IHD*	49		685		0.11	762	(3%)
		*Stroke*	6		79		0.15	91	(<1%)
		*Hypertensive diseases*	5		57		0.12	64	(<1%)
**Total (adverse effects)**			**650**		**19,244**			**26,610**	
Physical activity	IHD, ischaemic stroke, diabetes, breast cancer, colon cancer (30–64 years)	Current use of active transport to work (current health savings)	−40		−1,429			−*1874*	
		*IHD*	−*30*		−*1043*			−*1216*	
		*Ischaemic stroke*	−*1*		−*25*			−*43*	
		*Diabetes*	−*3*		−*115*			−*355*	
		*Breast cancer*	−*3*		−*131*			−*142*	
		*Colon cancer*	−*3*		−*115*			−*119*	
**Total (net)**			**610**		**17,815**			**24,736**	

Note: Data for road traffic noise refers to 2011, for the five main cities in New Zealand. Percentages are out of total attributable burden, excluding the health burden saved from walking and cycling. IHD = ischaemic heart disease. Numbers do not tally to 100% because of rounding.

These statistics disguise a marked variability in the risks associated with different transport modes, with motorcycles by far the most dangerous (See Appendix Table A1 for more details). Calibrating against data from the New Zealand Household Travel Survey [12], for example, we see that motorcycles had a death rate of 125 deaths per 1 billion km travelled, compared to only 5.6 for car and LGV users. Cyclists (25.8) and pedestrians (40.7) also had proportionately high death rates, while HGV drivers had the lowest risk (3.8). In terms of travel time (perhaps a more informative basis for comparison), the death rate for motorcyclists was 8.33 per million hours travelled, for cyclists 0.32, car and LGV users 0.21, pedestrians 0.16 and HGV drivers 0.14. In the case of injuries, motorcyclists and cyclists have by far the greatest risks (190 and 32 injuries per million hours respectively, compared with 3.5–9.3 per million hours for other modes).

Air pollution accounted for 283 deaths (44 per cent of the total), most of which (77 per cent) derived from PM_10_ rather than nitrogen dioxide. Deaths tend to be concentrated in the elderly, with the result that the combined effects represent only 4449 years of life lost, about 23 per cent of the total.

On the basis of our estimates, road traffic noise is responsible for a smaller health burden in New Zealand, accounting for 59 deaths from ischaemic heart disease, stroke and hypertensive diseases, and an equivalent of 821 years of life lost.

Physical activity through regular active transport for commuting also has a relatively small, though beneficial effect on health. Based on results of the Household Travel Survey, an estimated 346,000 adults in New Zealand are estimated to cycle or walk to work, of whom about 217,000 are aged 30–64 years (New Zealand Census 2013). Total distance travelled for these trips is an estimated 2010 million km/year. Based on the assumption that active transport currently raises these adults from low to moderate activity, this represents an estimated saving of about 40 deaths and 1429 years of life lost per year.

### 4.2. Results by Transport Mode

As has been noted above, allocating injuries and deaths to the underlying transport mode of cause is hampered by the way statistics are recorded and ambiguities in the case of multi-mode effects (e.g., accidents, physical activity). The results presented in Table 3 consequently refer only to deaths and need to be interpreted with care.

In terms of accidents, HGVs were involved in 52 of the road deaths (10 HGV occupants and 42 other road users) [50], representing 17 per cent of the total. With 50 deaths attributed to motorcycles, the remainder (206) can be tentatively attributed to cars, LGVs and buses. Deaths due to air pollution are likewise attributed mainly to emissions from cars, but because of their higher emission factors, both LGVs and HGVs account for a disproportionate number of deaths. Because of the lack of vehicle-specific emission factors, deaths associated with noise have been allocated proportional to distance travelled, and are thus mainly associated with cars. This, however, almost certainly underestimates the contribution from HGVs, which typically have considerably higher noise emissions.

**Table 3:** Approximate allocation of responsibility for deaths by transport mode, 2012.

Transport Mode	Billion Vehicle km Travelled (% total) ^2^	Estimated Number of Attributable Deaths, by Source ^1^					
		Traffic Injuries	PM_10_	NO_2_	Noise	Total (%)	Deaths per Billion Vehicle km Travelled
Cars	30.6 (74.3)	172	94	28	46	340 (52.3)	10.7
LGV	6.1 (14.8)	33	61	18	9	121 (18.6)	19.8
HGV	2.6 (6.3)	52	60	18	4	134 (20.6)	51.5
Buses	0.2 (0.5)	1	2	1	0	4 (0.6)	20.0
Motorcycles	0.4 (1.0)	50	1	0	0	51 (7.8)	127.5
**Total**	**40.0**	**308**	**218**	**65**	**59**	**650**	**16.3**

^1^ Table excludes deaths averted by physical activity; ^2^ Data from New Zealand Household Travel Survey [12].

Overall, the results show that cars are responsible for the majority of deaths (52 per cent), with HGVs accounting for 21 per cent and LGVs about 19 per cent. Expressed in terms of distance travelled, the highest risk is associated with motorcycles (127.5 deaths per billion km travelled), almost all from injuries. HGVs also account for a disproportionate burden of death (51.5 deaths per billion km travelled), over three times the risk associated with cars. Buses come out relatively badly in this analysis (20 deaths per billion km travelled), but this ignores the fact that these are multi-occupant vehicles, so that the number of person (as opposed to vehicle) kilometres is considerably greater. Cycling and walking are the only modes that can be considered directly to protect health, and account for the 40 deaths avoided as a result of increased physical activity.

### 4.3. Uncertainties

Uncertainties were evident throughout the rapid assessment, and arose from many sources. Key issues included errors in exposure assessment, largely due to inadequate monitoring data, uncertainties in the exposure-response functions and relative risks used to quantify health impacts, and gaps in knowledge that meant that some potential impacts had to be ignored. Reflecting these factors, uncertainties varied substantially between the different pathways, such that some were better estimated than others. Results of sensitivity analyses conducted to explore these uncertainties are shown in Appendix Table A2.

In the case of road accidents, we have a very high level of confidence in the mortality results, for the estimates are based on routinely reported data, gathered under strict protocols. As with all the pathways considered here, however, uncertainties arise in translating deaths and injuries into years of life lost and, more specifically, into disability-adjusted life years: lacking information on severity, we estimated the latter using the ratios of YLD to YLL derived from the Global Burden of Disease study [58]. Accident rates also vary substantially between years, though in New Zealand as in most developed countries accident rates are falling. Data for 2012, however, are close to the average of the surrounding five years (Appendix Table A2).

For air pollution, the uncertainties are greater. Substantial errors may have occurred in the exposure estimates because of the limitations of the available monitoring data and the simplistic models that were used to estimate the contribution from road traffic [17]. The effect of these cannot be easily quantified, but by analogy with other studies using regression-based air pollution models [66] the errors might be expected to be within the range of 20–50 per cent. For PM_10_, we also assumed that the contribution of motor vehicles to total concentrations remained steady at 22% from 2006 to 2012. Using different assumptions would have altered the estimated disease burden. For example, a reduction of the traffic-related contribution to 15% of the total, or an increase to 30%, would give a total of 150 and 300 deaths, respectively, compared to the original estimate of 218 (Appendix Table A2). For NO_2_, we reduced estimated impacts by 33 per cent to reflect possible overlap with particulates; different factors to allow for overlap would have changed the estimates proportionally.

Uncertainties are also inherent in the exposure-response functions used to translate the air pollution exposures into estimates of disease burden. These uncertainties are often reported as confidence intervals (typically 95 per cent) around the median risk estimate, both in the original studies and subsequent meta-analyses. An indication of their potential effect on the estimated burden of disease can thus be obtained by substituting the upper and lower confidence limits into the models. Here, this gives a plausible range of 150 to 300 deaths (compared to best estimate of 218) for particulates, and 37–94 deaths (best estimate 65) for nitrogen dioxide (Appendix Table A2). Taken together, these various considerations give us only moderate confidence in the estimates of disease burden from air pollution.

Assessment of the health burden from road traffic noise involved larger uncertainties. Due to the lack of data, exposure estimation had to be based on simplistic models, which could not be validated against measured noise levels. Analysis was also done only for the five main cities, so potential exposures in other areas (e.g., along the major state highways) were ignored, though the numbers of highly exposed people is likely to be small. For this reason, our results probably underestimate the numbers of people exposed to traffic noise. Exposure-response functions for the effects of noise are also less well defined than those for air pollution, and have larger confidence intervals. Sensitivity analysis using the 95% confidence limits (Appendix Table A2) thus gave a range of 18–177 deaths (best estimate 40). Additional uncertainties are also likely in transferring the exposure-response functions from Europe to New Zealand, because of differences in environmental conditions and population characteristics, while no allowance was made for possible overlap or interaction between noise and traffic-related air pollution [34]. For these reasons, we have low confidence in our estimates of the disease burden from noise. More research, including purpose-designed noise modelling and meta-analyses would be needed to improve this situation.

Assessment of the health impacts associated with physical activity were based on relatively well-established estimates of the health benefits from physical exercise, but involved major assumptions about the activity levels associated with walking and cycling. Only routine trips to work were considered, and all trips were assumed to be sufficient only to raise people from a low to moderate activity level (equivalent to 30 min of brisk exercise per day). Given that the average return trip duration for walking is about 40 min, and that for cycling, about twenty minutes, this assumption seems appropriate, though a proportion of people are likely to make substantially longer trips. It should also be noted that there is a marked discrepancy between estimates of the numbers of people commuting to work derived from the Household Travel Survey (used here) and the national Census. The latter reported a total of *ca.* 150,000 travelling by bicycle or car, compared to our estimate of 346,000. Using the Census data reduces the number of deaths averted to 17, and the YLL to 599 (see Appendix Table A2). On the other hand, both walking and cycling are done for other reasons than the journey to work—albeit often less routinely. Social trips, for example, are of a similar order of magnitude (166 million km/year). At maximum (assuming no overlap with commuters), these might raise an additional 150,000 people into the moderate activity level (see Appendix Table A2), and in that case would increase the health saving to 58 deaths/year. As a consequence of these uncertainties, we have low-moderate confidence in the estimates of the health benefits associated with physical activity, though overall it seems likely that they under-estimate the true effects.

Finally, the analysis omitted several potential effects of road transport. These include the acute effects and morbidity from particulates and nitrogen dioxide, due to a lack of readily available data to calculate DALYs for these. Other potential effects omitted from the analysis included the effects of secondary particulates, ozone and other traffic-related air pollutants, such as carbon monoxide, benzene and other volatile organic compounds (VOCs). The impact of noise on quality of life, cognition and subclinical symptoms of stress have also been ignored, as have potential effects due to emissions of greenhouse gases that contribute to climate change. Likewise we have not considered any potential intergenerational impacts, such as the effects of air pollution and physical inactivity on birth outcome and subsequent health. Given these exclusions, our results are likely to underestimate the overall health burden from road transport.

Uncertainties also occur in the attribution of the estimated effects to source modes. Because of the rules applied in attributing accidents to mode, our estimates may be slightly biased against HGVs and motorcycles, and in favour of walking and cycling (which were considered ‘innocent’ in terms of causing accidents). On the other hand, attribution of the effects of noise proportional to distance travelled is likely to have penalised cars and LGVs relative to HGVs, buses and motorcycles, all of which tend to have higher noise emissions. Overall, therefore, we have moderate to high confidence in our estimates of the proportional contribution to different modes.

More generally, it needs to be noted that this assessment was based on a conceptual framework developed by the authors, without input from stakeholders. Involvement of stakeholders (including those at risk from the impacts as well as policy-makers) is an important precept of IEHIA. Because of their personal experiences, stakeholders often bring a different perspective to any discussion about health-related risks, and often highlight issues that otherwise would be ignored, but to be effective the process of involvement is often relatively lengthy [67]. The topic of road transport, however, has been frequently discussed as part of other health impact assessments, and is well-documented. We therefore believe that the scoping of the issue done here, and illustrated in Figure 1, captures most of the factors of concern.

## 5. Discussion

### 5.1. Key Findings

The findings presented above show that road transport has a marked effect on public health in New Zealand, accounting for a net annual toll of *ca.* 17,815 years of life lost, and an estimated 24,736 years of healthy life lost (DALYs). In total, this represents at least 2.1 per cent of deaths in the country, and 3.3 per cent of the total years of life lost in 2012.

Road transport injuries, physical activity and traffic-related air pollution comprise the main agents of impact. On the basis of the data available here, road traffic noise appears to be of lesser importance, though our assessment may underestimate these effects.

In terms of mode, cars are responsible for about 52 per cent of the overall health burden, HGVs 21 per cent, LGVs 19 per cent, motorcycles 8 per cent and buses 1 per cent. Together, these demonstrate that HGVs and motorcycles make a disproportionate contribution to the burden of disease. Motorcyclists are also by far the most at-risk group, with death rates some 35 times those for car occupants, both per kilometre and per hour travelled. Cyclists, also, have a somewhat raised risk, for deaths twice that of car drivers and pedestrians, and for injuries (and DALYs) more than six-fold higher.

### 5.2. International Comparisons

In order both to evaluate the credibility of our results, and to put them into a wider context, the estimates of attributable disease burden from our study can be compared with those from other countries. In the case of traffic accidents, this is straightforward, because road safety data are routinely collected by most countries according to established, international protocols. For traffic noise, air pollution and physical activity, comparisons are much more difficult due to the limited number of published studies, and differences in the assessment dates, data quality and methodology of the studies that have been done. For all comparisons except physical activity, data are compared as DALYs per million people, in order to allow for differences in population size.

In terms of road accidents, it is evident that New Zealand performs rather poorly compared to other developed countries. In an environmental burden of disease study that closely mirrors our own, Kjellström *et al*. [43] produced estimates for Sweden in 2001 that translate to a figure of 2790 DALYs per million, considerably lower than ours (4752 DALYs per 1 million, for 2012). In the Netherlands, Knol and Staatsen [68] estimated that road traffic accidents were responsible for 5000 DALYs per 1 million people in the year 2000. Given that road safety has generally improved in recent years, the burden of disease attributable to traffic accidents has probably declined in both countries, and is now likely to be lower than that in New Zealand. This is supported by data published by the International Transport Forum [16]. In 2010, for example, New Zealand had the ninth highest road death rate from accidents (8.6 deaths per 100,000 people) out of 32 OECD member states. Expressed in terms of distance travelled, it was the third worst of 22 reporting countries (9.4 deaths per billion vehicle kilometres travelled). Data from the 2010 Global Burden of Disease Study [69] likewise show that New Zealand ranks amongst the worst of the developed countries, with rates of death from road vehicle injuries similar to those of many East European countries, Egypt, India and the USA.

For noise, data are much sparser and almost all the available studies relate to European countries. Knol and Staatsen [68] estimated a total of *ca.* 1000 DALYs per 1 million people for the Netherlands in 2000, some five times our equivalent for New Zealand (205 per million). For Switzerland, Vienneau *et al*. [49] give a rate of 603 DALYs per million people for cardiovascular mortality due to road traffic noise in 2010, while for Sweden Kjellström *et al*. [43] reported a figure of 457 DALYs per 1 million people due to hypertension and ischaemic heart disease. In a six country study (covering Belgium, Finland, France, Germany, Italy and the Netherlands) by Hänninen *et al*. [70], estimates of the disease burden from traffic noise in 2004 ranged from 371 DALYs per million in Finland to 1483 per million in France. Stassen *et al*. [71] estimated a burden of 3420 DALYs per million people for traffic noise in Flanders, Belgium, for the year 2004, some seventeen times our estimates, though this analysis included a wider range of morbidity effects, including sleep loss, annoyance and hypertension, for all of which exposure-effect relationships are relatively uncertain.

While many studies have estimated the health impacts of air pollution at national level (e.g., [68,70]), relatively few have attempted to separate out the contribution from road traffic. In Sweden, however, Kjellström *et al*. [43] estimated a total of 3970 DALYs per million people from PM_2.5_ and NO_2_ for 2001, four times our figure for New Zealand (995 DALYs per 1 million). In Switzerland, Vienneau *et al*. [49] used dispersion modelling to estimate exposures to traffic-related PM_10_. This implied a total burden of 1538 DALYs per 1 million people, about twice our equivalent for New Zealand (766 per million). An estimate can also be derived for Australia from the study of the health impacts of road traffic pollution by the Bureau of Transport and Regional Services [72]. This yielded a total of 900–2000 deaths due to motor vehicle-related air pollution in the year 2000. Using a base population of 19.2 million, and assuming twelve years of life lost for every premature death, this gives 552–1248 DALYs per million, a range that encompasses our own for New Zealand.

Although a large number of exploratory studies and prospective assessments have been done, comparable analysis of the health impact of physical activity associated with prevailing road transport use in real-world settings are sparse. However, relatively large savings in the burden of disease, especially from ischaemic heart disease and stroke, have been claimed for a switch from car to cycling use, in prospective assessments of road transport policies. Using the HEAT model, for example, Lindsay *et al*. [20] estimated a saving of 20.5 deaths annually through increased physical activity if 1 per cent of short car trips (*ca.* 45 million km) in urban New Zealand were switched to cycling, and 116.5 deaths from a 5 per cent switch (223 million km). This represents 1 death saved per 2.2 million cycling km and 1 per 1.9 million km, respectively. In Adelaide, using a comparative risk assessment approach similar to our own, but including a wider range of health outcomes over the age range 15+, Xia *et al*. [73] estimated a saving of 1 death per 4 million cycling km for a scenario yielding a 5 per cent switch to cycling and 1 per 3.75 million km for a 10 per cent switch. In sensitivity analyses, however, the number of deaths saved more than halved when the 70+ age range was excluded (implying a saving of approximately 1 death per 8 million km). In Barcelona, Rojas-Rueda *et al*. [74] estimated a saving of 12.46 deaths as a result of the introduction of a bicycle sharing scheme, which attracted 25,426 new users, cycling a total of 45 million km annually. This translates to 1 death saved per 3.5 million km cycled. In our study, by comparison, 40 deaths are estimated to have been avoided from 346,000 cyclists and walkers, travelling approximately 173 million km—equivalent to 1 death saved per 8650 people using active transport, and 1 per 4.3 million km walked or cycled.

These comparisons show distinct differences between our results in New Zealand and those from other studies and countries. In terms of road accidents, the differences are small, but the attributable burden in New Zealand is relatively high. In terms of air pollution and noise (and perhaps physical activity), the differences are larger, and the estimated impacts lower in New Zealand than the comparison countries.

These differences are no doubt due in part to differences in methodology between those used in this rapid assessment and those applied in more detailed studies. For example, our study used relatively simple modelling techniques for air pollution and noise, rather than the dispersion or geostatistical models used in many other assessments. Monitoring data in New Zealand are also sparser than those available to many other studies. Likewise, in assessing the effects of active transport, we used a simple assumption about changes in activity levels, rather than modelling energy expenditure, as often used in studies of cycling, and only considered regular commuting. Together, therefore, these comparisons confirm our suspicion that, though our results are of the correct order of magnitude, they tend to under-estimate the true health impact of road traffic in New Zealand, and should therefore be interpreted as low-end estimates of the attributable health burden.

Nevertheless, some of the differences may be real and reflect New Zealand’s specific geography. Compared to most of the other countries for which data are available, for example, New Zealand has a small and sparsely distributed populated. While traffic volumes are relatively high on a *per capita* basis, therefore, both traffic and population densities (per km^2^) are low, and relatively few people live close to busy roads. The potential for exposure to air pollution and traffic noise is, therefore, less. In the context of these differences, the relatively large burden in New Zealand from road traffic accidents presents an anomaly in our results. Given that data in this area are internationally consistent, this cannot be dismissed as an artefact of the methodology. Instead it implies something particular about New Zealand roads, vehicles or driving behaviour that raises accident risks above those of most other developed countries.

### 5.3. Policy Implications

Notwithstanding their inherent uncertainties, the results of this assessment clearly have implications for policy on transport and health in New Zealand. They not only show that road traffic creates a substantial public health impact, but also that road traffic accidents make a major contribution to the burden of injury and disease. Within this burden, motorcyclists and, to a somewhat lesser extent, pedal cyclists face particularly high risks. The results also suggest that heavy goods vehicles bear a disproportionate source of responsibility for the associated health impacts and deserve special attention.

The integrated framework used in this assessment also indicates the close interdependence of these health impacts, and hints at the direction in which policy needs to move. Piecemeal solutions, aimed at individual elements of the system, are rarely likely to be effective in totality. In some cases, they may even be counter-productive by transferring risks from one area to another, or by increasing the impacts of one pathway in order to reduce those from another. In the face of a growing population and rapid urban development (especially in the Auckland area), the real need is therefore for more integrated actions, which work coherently across the system.

The best way of achieving this is likely to be through interventions that shift a substantial proportion of the population towards walking, cycling and public transport, and away from reliance on car use. This would not only reduce emissions of air pollution and noise but also help to raise levels of physical activity, and thereby save further lives. The opportunity benefits of such policies have already been demonstrated by Lindsay *et al*. [20]. They suggested that a 5 per cent reduction in vehicle kilometres as a result of moving short trips (<7 km) from car to cycling could save as many as 117 deaths annually, as a combined result of reduced air pollution and increased levels of physical activity—and allowing for a small increase in deaths of cyclists in road accidents. Large savings in hospital admissions and restricted activity days due to injuries and pollution-induced illness were also indicated. Notably, the benefits in terms of reduced air pollution were small; the main health savings come from changes in physical activity. It is also apparent that additional benefits could be achieved by encouraging people to take public transport, as this usually involves some walking to access the system. Since New Zealand has a relatively high proportion (30 per cent) of people defined as obese [55], and tackling obesity is a major policy goal, such changes would have important social, as well as health consequences, far beyond the transport sphere.

### 5.4. Developing Environmental Health Indicators

Rapid assessments such as the one done here are not only useful in highlighting priorities and opportunities for policy action. They also help to show the information that is needed to support such actions in the longer term. Environmental health indicators are an important tool in this context [75,76], and over recent years a number of environmental health indicator sets have been established, both nationally and internationally [77]. To be truly effective, indicators need to be both comprehensive and balanced, in order to avoid biasing policy towards specific areas. If they are to guide policy in ways that minimise adverse health impacts, they also need to be focused on, and give direct estimates of, the scale of health effects. There is, however, little evidence that any of the indicator sets developed to date really match these requirements; most seem to be somewhat arbitrary, and driven largely by practical constraints of data availability and past or prevailing policy concerns. In the road transport domain, for example, indicator sets tend to focus on source activity (e.g., vehicle numbers, flows) and environmental pressures and proximal effects (e.g., concentrations of air pollutants) rather than health impacts. Where health outcome indicators have been included, it has often been in the form only of traffic accidents, or general measures such as overall respiratory mortality. The European Environment Agency [78] lists 17 transport-related indicators, of which five relate to atmospheric emissions and air pollution, and one to wellbeing (exposure to traffic noise). In New Zealand, the Ministry of Transport [79] maintains *ca.* 175 indicators, but of these only three are categorised as referring to public health: annual nitrogen dioxide concentrations, Auckland light vehicle emissions and noise measurements—and the last of these is void. In neither case, therefore, are any measures of health outcome reported. Innovatively, however, the recently published Air Domain Report for New Zealand [19] does include an indicator on estimated health impacts from human-made PM_10_.

The analysis done here shows the way in which a more rigorous, health-focused approach to indicator development can be achieved, by linking them to an integrated health impact assessment. On this basis, the conceptual model used to define the assessment (Figure 1) gives a framework within which to identify the indicators that are needed. The results likewise help to interpret the indicators, by showing the overall health burden that they represent, and the relative contribution attributable to each pathway and source.

This approach may seem daunting. In the case of road transport in New Zealand, for example, it implies indicators for each of the boxes in Figure 1—far beyond the small collection of “core” indicators often anticipated by policy-makers. The benefits, however, are substantial, for it ensures that the indicators provide a complete and balanced description of the system as a whole, and helps to reduce biases in decision-making. By structuring the indicators within a coherent framework, it also makes it easy to aggregate the information to a smaller number of “headline” indicators (e.g., total health burden from road transport) without distorting the information, or equally to drill down from any higher level of indicator to reveal the underlying causes and impacts.

Like other forms of information, however, indicators require data, and this analysis has also shown some of the problems that exist in this respect. Much of the monitoring needed to carry out the assessment, and to track health impacts from road transport, are currently deficient. Examples include the lack of measured data on ambient traffic noise, and the sparse and inconsistent coverage provided by the air pollution monitoring networks (including the lack of data on PM_2.5_). Opportunities for modelling, too, are limited by lack of relevant input data. In the case of noise, for example, modelling could be used to produce noise maps like those available for large urban areas in Europe, under the Environmental Noise Directive [80]. Similarly, dispersion modelling could be used to estimate exposures to air pollution. To allow this improvements are needed in the availability of georeferenced data, especially on traffic flows, building heights and emissions.

The issue of georeferencing also deserves emphasis. Geographically aggregated (e.g., national) indicators are rarely sufficient as a basis for assessing and tracking health impacts, as marked variations in exposure and risk typically occur across the community, often at a local level. Without information on where risks occur, it is impossible to identify who is at risk and thus to allow for the social factors that may be involved, either in assessing impacts or in developing effective policy responses. For example, evidence suggests that areas of higher socioeconomic deprivation bear the brunt of many of the impacts considered here—the so-called environmental justice effect [81]. Many of these effects are currently difficult to quantify because of a lack of suitably localised (or population-specific) data on the risk factors of concern.

In summary, a major constraint on indicator reporting and policy support in relation to road transport and health in New Zealand is the unavailability or limited quality of the relevant data. As is often argued, jurisdictions measure what they manage, and manage what they measure. The effect is to reinforce existing policy concerns and risk neglect of new and emerging issues. If policy is to be proactive and preventative, as is needed to avoid unwanted public health impacts, then it must have access to much more insightful information than is currently the norm—and this requires an underpinning of reliable, routinely monitored data.

Herein we see the value of the type of rapid assessment done here. Whilst completed quickly and at low cost (the total staff time for conceptualisation, data collation and analysis was *ca.* 300 hours), it provides a first approximation of the health impacts, attributed to source, which of itself can inform the policy debate. It provides, therefore, information for action in a timely manner, and gives a framework within which to identify the information needed for continuing policy support, and the indicators that should be developed for this purpose.

## 6. Conclusions

This study has demonstrated a working example of a rapid environmental health impact assessment, based on the IEHIA approach. The results suggest that road transport in New Zealand is responsible for a total of 650 deaths annually, mainly through traffic accidents and air pollution. Whilst impacts of air pollution and noise are low compared with other countries, those from accidents are relatively high, indicating the need for more effective policies to reduce accident risks. With a disproportionate contribution associated with HGVs and motorcycles, the results also indicate modal sectors that need specific policy attention. The results also show the public health gain provided by active transport, which currently saves an estimated 40 lives annually. This further highlights the public health importance of an increased use of public transport, cycling and walking as forms of transport in New Zealand, rather than the current heavy reliance on cars.

This example also shows the value of conducting rapid assessments of this type. By making use of readily available data and approximation techniques, they provide a quick, low-cost quantification of the health burden associated with both simple and more complex policy issues. While the results must be recognised as inexact, they nevertheless provide important evidence to inform the policy debate, and help to define a comprehensive and balanced set of indicators for policy support. The process of undertaking the assessments also highlights the strengths and weaknesses of existing monitoring systems and data.

## Appendix

**Table A1:** Traffic accidents.

Transport mode (Victim)	Distance (bkm)	Hours (Million)	Deaths	Injuries	Deaths (%)	Injuries (%)	Deaths/1b km	Deaths/1m h	Injuries/1b km	Injuries/1m h
HGV	2.6	70	10	246	3.2	2.0	3.8	0.14	94.6	3.5
Car/LGV	36.7	991	207	8975	67.2	74.3	5.6	0.21	244.6	9.1
Motorcycle	0.4	6	50	1138	16.2	9.4	125.0	8.33	2845.0	189.7
Cyclist	0.31	25	8	798	2.6	6.6	25.8	0.32	2574.2	31.9
Pedestrian	0.81	205	33	917	10.7	7.6	40.7	0.16	1132.1	4.5
Total/Average	40.8	1297	308	12,074	100	100	7.5	0.24	295.8	9.3

Data on accidents derived from: Ministry of Transport *Motor vehicle crashes in New Zealand 2014*. Available online: http://www.transport.govt.nz/research/roadcrashstatistics/motorvehiclecrashesinnewzealand/motor-vehicle-crashes-in-new-zealand-2014 Accessed on 1 September 2015; Data on distance and hours travelled derived from: Ministry of Transport *Comparing travel modes. New Zealand household travel survey 2011–2014*; New Zealand Government: Wellington: 2015; Data for cars/LGV and HGVs estimated by applying average speed for car drivers, derived from the above, to distance data in previous source.

**Table A2:** Sensitivity Analysis.

Pathway	Assumption in Best Estimate	Alternative Condition	Attributable Deaths
Road accidents	Baseline scenario		308
	Use traffic accident statistics for 2012	Use traffic accident statistics, averaged over 5 years (2010–2014)	303
Particulate matter (PM_10_)	Baseline scenario		218
	Assume that the proportion of deaths attributable to motor vehicles has remained at 22% from 2006 to 2012	Use a lower proportion for motor vehicles than in 2006 (15% attributable)	150
		Use a higher proportion for motor vehicles than in 2006 (30% attributable)	300
Physical activity	Baseline scenario		−40
	346,000 people currently commuting by active transport	150,000 people currently commuting by active transport (2013 Census)	−17
		500,000 people raised to moderate activity as result of combined effects of routine commuting (346,000 people) and social trips (140,000) by active transport	−58
Nitrogen dioxide	Baseline scenario		65
	Relative risks/exposure-response function—best estimate used	Use lower and upper bounds of relative risks	37–94
Road traffic noise	Baseline scenario		59
	Relative risks/exposure-response function—best estimate used	Use lower and upper bounds of relative risks	12–80
